# The New York Institute of Stomatology

**Published:** 1900-10

**Authors:** 

**Affiliations:** The New York Institute of Stomatology


### Reports of Society Meetings.

### THE NEW YORK INSTITUTE OF STOMATOLOGY.

A REGULAR meeting of the Institute was held on Tuesday, May 1, 1900, at the office of Dr. Louis Shaw, No. 162 Remsen Street, Brooklyn, New York City, the President, Dr. E. A. Bogue, in the chair. The minutes of the previous meeting were read and approved.

### COMMUNICATIONS ON THEORY AND PRACTICE.

*Dr. Louis Shaw.*—I wish to present to the Institute some sets of teeth which were made in Japan; they may be of interest to the members of the society as showing the progress of dentistry in the far East.

*Dr. S. E. Davenport.*—In these days of quack nostrums and secret filling-materials, it is a pleasure to feel this body stands for the opposite. I believe we prefer to know something about the filling-materials we use and the remedies we prescribe rather than to take them altogether on faith. As scientific men, I think we should not allow ourselves to use anything the component parts of which we are not familiar with.

In that connection I should like to refer to an amalgam which I have used with satisfaction for a year and a half. It is made by a dentist, Dr. Bement, of Lockport, New York. Upon each package Dr. Bement prints the formula from which the alloy is made. It is sold at a very low price, five ounces for six dollars, which Dr. Bement claims is quite sufficient for any alloy of those metals, leaving him all that a manufacturer's profit should be. I find this amalgam of good color and possessed of good working qualities. Of course, the length of time I have used it is too short for me to make any positive statements as to its durability. I have thought it a privilege to call attention to this amalgam, which is made, and well made, by a dentist in this open and scientific way.

*Dr. C. B. Parker.*—Is it a quick-setting amalgam?

*Dr. Davenport.*—Sufficiently so.

*Dr. J. Morgan Howe.*—I should like to endorse what Dr. Davenport has said with regard to this particular alloy. I have used it with satisfaction for over a year, and the fillings which I first put in with it appear very well to-day. I was induced to use it because the formula is published on the package, and is similar to other alloys that have proved good in use. Dr. Bement has informed me that he would be happy to make an alloy from any desired formula. In conversing with a *confrère* not long ago, he told me of having made alloys from formulæ of which he thought highly, and he suggested to me that it would be well for us to agree upon a formula that met with our approval and have our alloys made to order by Dr. Bement, or some other metallurgist, according to the prescription which we should decide upon. I think we should do this to supply any deficiency there is in the supply of alloys whose formulæ are made known.

*Dr. A. H. Brockway.*—Can Dr. Davenport tell us how near like Dr. Black's formula this alloy is of Dr. Bement's? Dr. Black's formula is nearly half and half silver and tin, with a small amount of gold.

*Dr. Howe.*—It does not contain nearly so much silver as Dr. Black's formula and many makes of the quick-setting amalgams with secret formulæ which have been rushed upon the market within the last year or two, but I have found by experience that alloys which do not contain nearly so much silver have made good fillings, and some such fillings put in so long ago as twenty-five years are showing well to-day.

*The President.*—Dr. Black, you know, does not trust to clinical experience alone. I am now having an assay made of an amalgam which has done me better service than any alloy I have ever used. I have used it for more than twenty years.

*Dr. C. O. Kimball.*—I should like to ask Dr. Davenport regarding Shattuck's amalgam, which I know he has used and recommended very highly. What are the relative merits of the two?

*Dr. Davenport.*—Dr. Bement's alloy works much like Shattuck's, but seems to be a finer-grained and cleaner amalgam.

*Dr. Howe.*—I wish to call attention, in a very informal way, to the use of eugenol and oxide of zinc, mixed, as suggested by Dr. S. Blair Luckie, of Chester, Pa., in 1898, in the July number of the *Items of Interest.* His paper seemed to be called out by the discussion of a proprietary article at a meeting of the New Jersey Society. Dr. Luckie states in his article of that date that a cement can be made by mixing these two substances together, which answers for a pulp-capping and a lining for deep cavities and a cement to fill cavities with, claiming that it would harden and last as well as the ordinary cements that we use for temporary fillings. I have not used it for this latter purpose, my experience having been confined to the covering of exposed or nearly exposed pulps with this mixture, and I have found it to work so well that I have thought it worthy of bringing to your attention. Dr. Luckie's directions are that the oxide of zinc shall be added to the eugenol and mixed as the ordinary cement until the mass becomes crumbly, when upon working it a little more with a spatula it softens again, admitting of the addition of a little more of the powder. In that way a mass can be made which softens again by kneading, and can then be placed in the bottom of a cavity. I have used it in that way in deep cavities to protect the pulp from the action of phosphoric acid, which it seems to have done very satisfactorily. My experience dates from the publication of Dr. Luckie's article, and I call your attention to it and suggest that it is worthy of a trial. I requested Dr. Luckie to give us the benefit of his further experience, and I am glad to say that he has responded very cordially. I have with me Dr. Luckie's communication, which I will ask the secretary to read.

(For Dr. Luckie's communication, see page 652.)

*Dr. Davenport.*—I would like to ask Dr. Howe whether he would consider it advisable to use oxide of zinc and eugenol in deep cavities in which he intended to place a gold filling,—does the combination set quickly enough to allow of the immediate introduction of gold?

*Dr. Howe.*—No, I should think it inadvisable. I have used it in some such manner where the cavities were deep and were to be filled with amalgam, first placing in the lining, then covering it with the oxyphosphate, and then introducing the amalgam.

*The President.*—Did you contemplate bringing the action of phosphoric acid under this discussion?

*Dr. Davenport.*—That would be quite pertinent, Mr. President, as it is the action of phosphoric acid from which this cement is to protect the tooth.

*The President.*—The gentlemen will kindly understand that the reference which Dr. Luckie made in his paper to the reason why he used this mixture is also before you for discussion. It is to be hoped that Dr. Howe will also give us some points thereupon.

*Dr. Brockway.*—There is a preparation which I have used, called jodo-formagen, which is sold at the dental depots and which seems to answer all the purposes which this mixture does. I have used it for over a year with very great satisfaction. It is used like a phosphate cement. It can be used in many cases where there is an actual exposure without any bad after-effects. I do not know that it is any better than the eugenol and oxide of zinc, but it is, I think, just as good.

I have also used a preparation supplied by Dr. Kellogg, of Peoria, for the same purpose, but the objection to it is that it is a vile-smelling compound. Our friend, Dr. Thayer, got out something very similar, for which he claimed remarkable results. I believe the claim is valid.

*Dr. Davenport.*—Will Dr. Brockway be kind enough to give us the constituents of these various remedies which he has told us about. Of course, Dr. Brockway would not use anything the component parts of which he was not familiar with. As I understand it, Dr. Luckie has experimented with eugenol and oxide of zinc, finding the combination valuable, and if we can get all the help we need from this substance which we know all about, it seems to me that we ought to use it in preference to the proprietary articles of which we know nothing.

*Dr. Brockway.*—I believe there must be iodine and formaldehyde in the jodo-formagen, as the name would indicate.

*Dr. J. A. Schmidt.*—It is composed mostly of iodine, with a small percentage of formaldehyde and some of the essential oils. It works very nicely, although I prefer to use it not anterior to the second molar, as it becomes green and may stain the tooth. It is an expensive article. If this preparation of Dr. Luckie's will answer the same purpose, it would be preferable from the fact that it is less expensive. I have used the eugenol and oxide of zinc as a capping, and found it worked very nicely. It was in years gone by when we used to cap pulps.

*Dr. F. Milton Smith.*—I once heard a clergyman addressing a body of clergymen relative to educational institutions, which he said must be thoroughly first-class if they were to obtain thoroughly first-class patronage. "Now," he said, "we are all strictly temperate here and would not drink water unless it was boiled." So we should not use any of these various remedies whenever it is possible to avoid it, unless we know their component parts. However, I have had the misfortune to be obliged to use some things of which I did not know exactly the composition. This remedy which Dr. Brockway has suggested is one of them. When he mentioned the name, the words of Dr. Davenport came to my mind. This cement has been of a very great convenience to me and I have, with Dr. Brockway, had apparently good success with its use. But it seems to me, if Dr. Luckie has anything which he is willing to tell to the profession, and which he has been some little time studying out, if it is anything like as good, we ought to give it the preference.

*Dr. Kimball.*—I should like to place myself on the side with Dr. Smith. I believe in Dr. Davenport's theory and Dr. Brockway's practice. In other words, I have used the jodo-formagen cement with some misgivings. I do not like to use anything the exact composition of which I do not know. They publish a formula which, so far as it goes, is reasonably satisfactory, but is not complete, so I use the preparation with some hesitation. I do believe most thoroughly in the wisdom, so far as possible, as formulæ are brought forward to us which have been carefully proved and of which we know the full detail, and which have been shown to be good, of using them to the exclusion of those preparations of which we are at all doubtful. I think our thanks are due to Dr. Luckie for having brought this before the profession.

*Dr. Davenport.*—"Dr. Davenport's theory" is not to me a pleasant expression. I do not purpose to pass as a crank in this assembly, nor to convey the impression that I do not use patented remedies. I do use some things the component parts of which I know little, but I use them only when I am obliged to, and as between such an article and one which has been presented in a professional way, particularly if prepared by one who is skilled in our profession and who knows our needs, I give the latter the preference without hesitation.

*Dr. Kimball.*—I am glad Dr. Davenport places himself squarely on the side with Dr. Smith and myself.

*The President.*—I regret there are no further remarks to be offered on this interesting subject. During the discussion I have made a note which I think might very profitably be made the subject of some future meeting. My question is, What should we do when obliged to treat certain roots in which no filling has been placed, but over which a filling has existed for years? Is the eugenol and oxide of zinc applicable here? and why?

*Dr. Davenport.*—Probably the successful dentist from whom we are about to hear can enlighten us.

*Dr. E. H. Raymond.*—It is with some hesitation that I present to this body a paper on such a subject as I have selected, as many of you are, and have been for years, eminently successful in the practise of your profession. There are, however, others whose eyes may fall upon the words to be uttered who may be helped by them. It is with this object in view that I ask your attention for a few moments.

(For Dr. Raymond's paper, see page 654.)

*The President.*—I think we have heard something which might warrant comment from every one present, and we owe our thanks to the essayist. We shall be pleased to hear from those who are willing to discuss Dr. Raymond's paper.

*Dr. Kimball.*—I have listened, as you all have, with great pleasure, and I wish to express to Dr. Raymond our thanks for his putting it so clearly, so simply, and so beautifully. The successful dentist in my judgment is the man, first and above all, who makes his own character the very best. That, in dentistry, as in other things, is the foundation, and Dr. Raymond rightly emphasizes the importance of the personal element in the case. I do not know of any profession where more is required of personal character than in dentistry. I was very much struck by the remarks which a lady made in my office a few days ago. She said, in a laughing way, but I know that she meant it, that somebody was advising her to go to so-and-so, but she said "No," that as far as her teeth were concerned she must go to a good Christian. She felt that she was going to a man whom she could trust, and so I say the predominant question in successful dentistry is the question of personal character. If we aim at high ideals of personal life, doing each day our very best, then we shall succeed.

*Dr. F. Milton Smith.*—I have been very much pleased with Dr. Raymond's paper. My thought has been for years that successful men could be of vast help to at least the younger men of the profession if every now and then they were to give them just such a paper as Dr. Raymond has given us to-night. Not exactly the same material, of course, but material taken from their experience. This thought has been more thoroughly emphasized in my own mind from the fact that my early experiences in a dental office were so barren of advantages. Personal character enters very largely, more so than most of us think, into the elements of a successful dentist. I believe there is no man in this world who ought to have the confidence of those who transact business with him more than the dentist. I know of no one, unless it is the plumber, who is able to steal their money as easily if he chooses, and, strange as it may seem, we run across a patient every little while who thinks nothing of disputing his bill, when, if he would only stop to think, he would see how easy it would be for us to have taken advantage of him in other ways. Regarding the instruction which comes from a successful man: The man under whom I made my first studies was a thoroughly good mechanic,—and Dr. Raymond has said that that is very essential; he could put in gold plugs that were splendid and saved the teeth beautifully, but when you have said that you have said about all. He knew nothing about handling patients, and had no system of charts or anything of that kind. When he had the dam on ready for a gold filling, it was nine chances to one he would call me from the laboratory and send me after the gold. This man never in his life received an income from his practice of more than fifteen hundred dollars a year. Dr. Raymond has referred to the question of care about the person, about the behavior, about being patient and sympathetic. I think in our profession we have the best possible school for patience. I do not know that I would care to go through life again without the lessons which I have learned in this way. Regarding personal cleanliness, an incident happened when I was just a boy, only a few months in my studies, which greatly impressed me. I was visiting a personal friend of mine practising in a city up the State. He was a splendid fellow, of elegant appearance, and a successful dentist. I think he was one of the finest specimens of physical manhood I have ever seen. I have to-day fillings in my bicuspid teeth which he put in twenty-five years ago. I arrived at his office just as he returned from lunch, and he proceeded to make his toilet. I think it took him nearly half an hour to get himself in proper shape for the afternoon's work. He had a very busy afternoon, seeing a number of patients, but he did not wash his hands from the time he started in till he finished. This fact impressed me most thoroughly. I shall always remember the remarks of the late Dr. Abbott, which he emphasized in his lectures to the class. "Gentlemen," he would say, "wash your hands every time a patient comes in, and let them see you do it." I believe there is a great deal in this. It is these little things which seem to be slight, but which create the first impressions upon the patient.

I am very glad of having the opportunity of listening to Dr. Raymond's paper.

*Dr. L. C. Leroy.*—I do not know, Mr. President, that I can say much more than has been said, except that I did not get a chance to say a word on the subject that is passed. I would like to take this opportunity to say that in spite of all the odors that might arise from iodoform, or any other drug that we may wish to use, if there is virtue in that drug that can be turned to our account we should use it. A few drugs, such as iodoform and trichloracetic acid, are of great value; however, I am very cautious in their use, and do not believe my office has been contaminated with their odors. I do not believe, as I have heard so many times, in discarding a drug simply on account of its fancied unpleasant odor. In the practice of general surgery many measures are adopted which are not agreeable, but which are effective and are not discarded because they are unpleasant.

*Dr. Davenport.*—We are under obligations to Dr. Raymond for the service he has rendered us to-night, and for what he has done for the readers of our proceedings. Dr. Raymond felt a little hesitation, I know, about presenting a paper upon this subject, but he was advised that we all thought, with him, that a great many young men who were perhaps following with interest the proceedings of these meetings would be benefited by a statement of the essayist's ideas on this subject. I think Dr. Raymond ought to be congratulated upon the facility with which he constructs sentences, as this is as smoothly written a paper as has been presented to the Institute for a long while. There is very little to be said in the discusssion of the paper except to commend it and to give it our cordial acceptance.

*Dr. Howe.*—I would like to express my gratification at hearing Dr. Raymond's paper. There is nothing which I can say in discussion, and I wish to express my appreciation to the doctor for having done so well what was set for him to do.

*Dr. F. Milton Smith.*—There is just one thought which Dr. Raymond brought out in the paper which might possibly lead some conscientious young man astray. As I understood him, he said, "Never charge for an operation unless it is successful." Afterwards he says, "Never warrant an operation." I believe that some young men, and older ones for that matter, do operations over for which no charge is made, which never should be done. If I had my life to live over again, the operations which I would do over without charge would be far fewer. An operation may be successful, and still not be successful in the eyes of the patient; and the young dentist, fearing his interests may suffer, loses his fee. Only a few evenings ago, in talking with one of our successful men, he spoke of having rendered a bill for some considerable amount for services. Before the bill was paid the patient came to him again, and on account of the breaking away of the tooth one of the teeth had to be refilled. He then sent another bill which included the last operation. As the bill was disputed, he told the patient that in the first bill he had charged for the very best services he was able to give under those conditions, using his very best judgment and skill, and under those circumstances, the filling having to be replaced, he was entitled for a fair remuneration for doing it. Of course, if the operator feels that the operation could have been done better, he should do it over again, but it is better to be careful the first time.

*Dr. Raymond.*—I had in mind, when I wrote the sentence in reference to making no charge for a failure in our operations, this fact: Sometimes, while filling a difficult cavity, no matter how careful one may be, some accident, such as moisture, may prevent perfect cohesion of the gold or adhesion of the cement if it be an inlay, and failure results. If within a year such a failure occurs, I do the work over without compensation. In regard to warranting an operation, I have found that it is not a safe thing to do. We do not know what chemical or mechanical changes may take place in the mouth.

*The President.*—I think our Executive Committee should be congratulated on the profitable matter they have presented to us this evening, and both the gentlemen deserve our thanks for their efforts. I trust that they will in a future meeting provide us with a discussion of the subject I have already mentioned,—namely, "What do we do when obliged to treat certain roots in which no filling has been placed, but over which a filling has existed for years?" We all know what happens when we open into such a tooth, unless we are extremely careful to open under antiseptics and to properly treat. Now, what is proper treatment? I think we could very profitably listen to a paper upon this subject.

Adjourned.

Fred. L. Bogue, M.D., D.D.S.,
*Editor The New York Institute of Stomatology.*

